# Femoral Bowing and Femoral Neck-Shaft Angle Evaluation Can Reduce Atypical Femoral Fractures in Osteoporotic Patients: A Scientific Report

**DOI:** 10.7759/cureus.10771

**Published:** 2020-10-02

**Authors:** Ioannis Papaioannou, Georgia Pantazidou, Andreas Baikousis, Panagiotis Korovessis

**Affiliations:** 1 Orthopedics and Traumatology and Spine Surgery, General Hospital of Patras, Patras, GRC; 2 Otolaryngology - Head and Neck Surgery, General Hospital of Patras, Patras, GRC

**Keywords:** atypical femoral fractures, bisphosphonates, femoral neck-shaft angle, femoral bowing

## Abstract

Bisphosphonates (BPs) are the mainstay of osteoporosis treatment due to their safety and efficacy. There is evidence that BPs medication may be complicated by atypical femoral fractures (AFFs). Prolonged administration of BPs is even more strongly associated with AFFs. AFF is a relatively rare complication of BPs when taking into account the huge population worldwide that benefits from this pharmacotherapy. AFF is, however, a serious complication of BPs treatment, which includes prolonged healing time and high revision rate when operative treatment is required. Less frequently, AFFs occur even without BPs administration, while these fractures have all the characteristics of “stress” or “insufficiency” fractures. The critical point of view in AFFs pathogenesis seems to be not only the biology of cortical bone, but also the mechanical issue. It has been proven that BPs, glucocorticoids and proton pump inhibitors (PPIs) can cause bone turnover suppression and affect the biological parameter of AFFs pathogenesis. Specific mechanical femoral bone properties predispose to AFFs pathogenesis. Several studies have already reported that increased femoral bowing > 5.25^0^ degrees or decreased femoral neck-shaft angle <125 degrees, are associated with increased risk for diaphyseal and subtrochanteric AFFs respectively, regardless of BPs uptake. If these two parameters are simultaneously present, the probability for AFFs occurrence increases dramatically. Our scientific report, which is based on the current evidence about AFFs, is that if both femoral bowing angle and femoral neck-shaft angle are evaluated before BPs administration, this intervention may reduce the incidence of AFFs. Thus, in cases with excessive lateral femoral shaft bowing or very small femoral neck-shaft angle, the prescription of another anti-osteoporotic treatment than BPs should be recommended. If, however, BPs can’t be avoided, clinicians should be aware of the fact that long-term administration may be implicated with AFFs occurrence. In these cases, short term BPs administration with timely drug holiday between three and five years may be reasonable. Finally, roentgenographic evaluation of both femurs every six months and medical reference in case of any emerging thigh pain are also logical interventions to prevent and reduce AFFs.

## Introduction

Atypical femoral fractures (AFFs) are often associated with bisphosphonates (BPs) administration for osteoporosis treatment [1]. As BPs are the most commonly prescribed and highly effective treatment in menopausal and senile osteoporosis, AFFs are considered as a serious complication of this anti-osteoporotic treatment. Additionally, when surgical treatment is required for AFFs, increased healing time and high revision rate is anticipated [2]. There is evidence that BPs administration consists of a risk factor for AFFs incidence [3], while prolonged administration of BPs is even more strongly associated with AFFs [4]. Few studies have already reported several cases of AFFs without BPs usage [5], while in 2013 the American Society for Bone and Mineral Research revised the definition of AFFs by deleting the clause pertaining to BPs usage or other drugs that influence bone turnover suppression such as proton pump inhibitors (PPIs) and glucocorticoids [6]. The pathogenic mechanism of AFF is not well understood, although there is evidence that the pathogenesis of AFF is associated with both mechanical and biological mechanisms of cortical bone [7].

## Technical report

As the prevalence of osteoporosis increases dramatically, AFF is a devastating complication of BPs usage, our scientific report, that is based on current evidence about AFFs, is that if both femoral bowing angle and femoral neck-shaft angle are evaluated before BPs administration it may reduce the incidence of AFFs. Thus, in cases with excessive lateral femoral shaft bowing or very small femoral neck-shaft angle, prescription of another anti-osteoporotic treatment other than BPs should be recommended. If, however, BPs can’t be avoided, clinicians should be aware of the fact that long-term administration may be implicated with AFFs occurrence. In these cases, short term BPs administration with timely drug holiday between three and five years may be reasonable. Patients with osteoporosis and increased BMI (body mass index) [5] should also be monitored for these angles, because due to mechanical reasons they are more vulnerable to AFFs. Finally, we recommend clinicians to evaluate these angles not only in patients with osteoporosis and BPs usage, but also in patients who consume drugs affecting bone turnover such as glucocorticoids or proton pump inhibitors. Glucocorticoid therapy, older age, increased BMI and decreased height are proposed as additional risk factors for AFFs by few studies [5,8], but further evaluation with large studies is mandatory.

## Discussion

AFFs belong to the category of insufficiency fractures as they present almost all the essential elements of them [6]. The mechanical pathogenesis of AFFs seems to be the failure of the lateral femoral cortex to withstand the increased tensile stress applied on it [9]. The most compound hypothesis for the healing of insufficiency fractures is that it occurs through osteocyte apoptosis and selective remodeling of the injured femoral cortical bone, that signals for bone healing by increased production of the receptor activator of NF-kB ligand, local osteoclastic activation, and bone formation by osteoblasts to replace the resorbed bone by osteoclasts [6]. The increased incidence of AFFs in patients receiving BPs or other medication that affects bone turnover can be explained by the deactivation of osteoclasts and suppression of the remodeling process that associates the administration of BPs. All these actions, negatively affect the repair of the insufficiency fractures in AFFs, allowing cracks to increase [6].

In cases with atypical femoral shaft fractures, the lateral femoral bowing plays a significant role in pathogenesis of these fractures [10]. AFF incidence and fracture location are strongly correlated with the degree of femoral bowing [9,11,12]. Femoral bowing is measured as the angulation between the proximal and distal quarters of the femoral diaphysis and the cut-off value was defined between 5.25^o^ and 7^o^ [9,12]. Clinicians shouldn’t be adhered only to the femoral shaft bowing angle, but also to the general profile of the patient and the risk factors for AFFs development. On the other hand, in patients with normal femoral bowing the weakness of the femur is located in the sub-trochanteric area, where bone strength is low due to the thin lateral cortex [8]. The distribution of mechanical forces in this area is absolutely influenced by the degree of femoral neck-shaft angle. Decreased femoral neck-shaft angle is associated with increased tensile stress to the thin subtrochanteric lateral cortex and subsequently vulnerability for AFFs [8]. This has already been confirmed by a study from Oh et al, in which the subtrochanteric AFFs are associated with a very small mean femoral neck-shaft angle (125,8^o^) [8].

It is worth noting that patients who develop AFFs are somewhat younger than those who develop osteoporotic proximal femoral fractures, with a mean age of 74-77 years old (AFFs group) [13,14].

As the population is getting older, bone mineral density decreases and prevalence of osteoporosis increases. BPs are the mainstay of osteoporosis treatment, so evaluation of femoral bowing and femoral neck-shaft angle can contribute to the reduction of AFFs either by no initiation in extreme cases, or by administration of BPs for a relatively shorter period of time than usually, introducing drug holiday timely.

**Figure 1:**
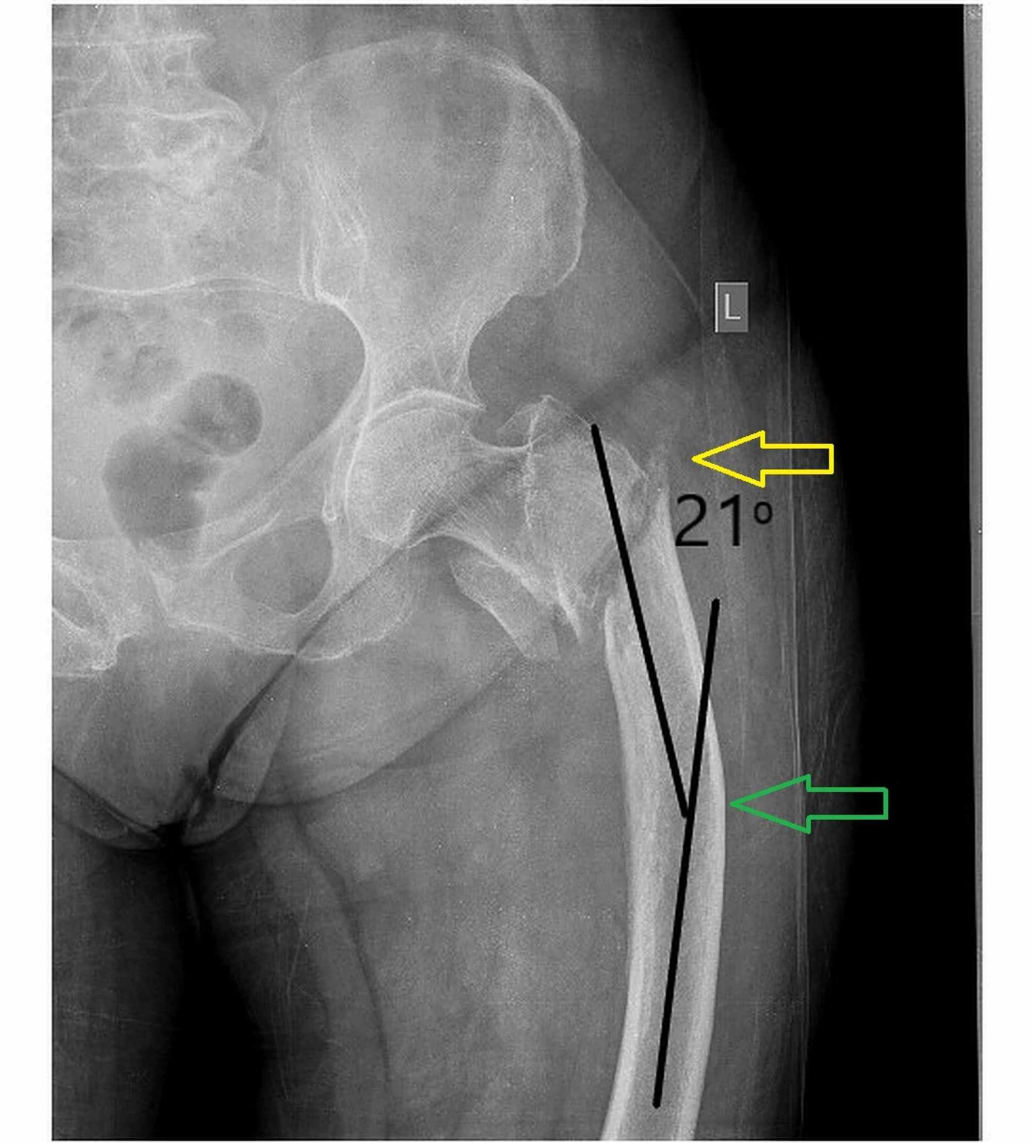
Anteroposterior x-ray of hip and proximal femur demonstrates intertrochanteric fracture and excessive lateral femoral bowing (21 degree). The yellow arrow shows the hip fracture, while the green arrow shows the femoral bowing.

To support our scientific report, we appose an example of a female patient, 85 years old with senile osteoporosis with intertrochanteric hip fracture and excessive lateral femoral bowing (Figure 1).

After fixation of the fracture, we should treat our patient for osteoporosis. As it has already been highlighted, AFF pathogenesis is associated with both mechanical and biological mechanisms of cortical bone [7]. In this case, the mechanical parameter is certainly affected by the excessive femoral shaft lateral bowing (21^o^). If, in this case, we prescribe BPs for osteoporosis treatment, we also affect the biological parameter and we simultaneously increased the possibility for AFF occurrence. In this particular case, the administration of an anabolic agent plus calcium and vitamin D is recommended [15]. Furthermore, we evaluated the contralateral femur for any incomplete AFF and we also consult our patient to be alert in case of any emerging thigh pain in future. Finally, we recommend roentgenographic evaluation of both femurs every six months [16].

It is worth noting that the incidence of atypical fractures is less significant than the benefit of BPs administration [17]. BPs administration reduces risk of hip, vertebra and other bone fractures as much as 50%-70% and undoubtedly these drugs are the mainstay of osteoporosis treatment based on their safety and efficacy [18,19].

The critical point of view is that AFFs have all the characteristics of stress or insufficiency fractures, and the pathogenesis of these fractures is affected by mechanical stresses and by cracks self-healing bone potency [6]. In cases of elderly (>70 years old), who visit physicians for osteoporosis evaluation and therapy, we propose radiographic evaluation of both whole femurs, to measure femoral bowing and femoral neck-shaft angle according to our scientific report. If the femoral bowing is >5.25^o^ or the femoral neck-shaft angle is < 125^o^, there is an increased risk for diaphyseal and sub-trochanteric atypical femoral fracture respectively, regardless of BPs usage [8]. So, if the mechanical parameter of AFFs pathogenesis is already affected and we prescribe BPs for osteoporosis treatment, we also affect the biological parameter of AFFs pathogenesis and the possibility for AFFs occurrence increases dramatically. In extreme cases with excessive lateral bowing or very small femoral neck-shaft angle, we recommend the prescription of another anti-osteoporotic treatment. In patients with increased lateral bowing (>5,25^o^) or small femoral neck-shaft angle (<125^o^), if BPs administration can’t be avoided, clinicians should be aware of the fact that long term therapy may be implicated with AFFs occurrence. In these cases, short term BPs administration with timely drug holiday between 3 and 5 years is reasonable. Thus, BPs administration for more than 5 years should be avoided and these patients should be counseled properly and followed up regularly. Few studies have shown that there is a tendency for AFFs to occur bilaterally [16], so follow up should include both femurs. In obese patients, the mechanical parameter becomes more crucial and administration of drugs that affects bone turnover should be further evaluated [5].

## Conclusions

To the best of our knowledge, proposal for femur radiographic evaluation before anti-osteoporotic treatment prescription has never been reported in the current literature, to reduce AFFs. Considering the large population benefiting from BPs pharmacotherapy, the incidence of this fracture entity is rather low. Despite that, we believe that with this scientific report, AFFs can be further reduced, although more research is urgently needed is in this area, to confirm this.
